# Systemic immune responses in patients with spine surgery related infections

**DOI:** 10.3389/fimmu.2026.1805889

**Published:** 2026-05-26

**Authors:** Pia Fehrenbach, Siegmund Lang, Maja Gocević, Puk Kwant, Daniel Haschtmann, Tamas F. Fekete, Ursula Menzel, Esther C. de Jong, T. Fintan Moriarty, Sibylle Grad, Markus Loibl

**Affiliations:** 1 AO Research Institute Davos, Davos, Switzerland; 2 Department of Experimental Immunology, Institute for Immunity and Infectious Diseases, Amsterdam UMC, University of Amsterdam, Amsterdam, Netherlands; 3 Universitätsklinikum Regensburg, Regensburg, Germany; 4 Schulthess Klinik, Zürich, Switzerland; 5 Center for Musculoskeletal Infection, University Hospital Basel, Basel, Switzerland; 6 Eidgenössische Technische Hochschule Zürich, Zürich, Switzerland

**Keywords:** immune cell profiling, immune cells, infection, spine, surgical site infections

## Abstract

**Introduction:**

Surgical site infections (SSIs) are significant complications that can occur after spine surgery. Compared with systemic infections such as sepsis, or localised musculoskeletal infections such as periprosthetic joint infection, the immune responses in postoperative infection of the spine are poorly understood. A deeper understanding may provide clinically relevant diagnostic or therapeutic options.

**Methods:**

This study examined changes in immune cell profiles in the blood of patients who developed infections after spinal surgery (Infection group, I, *n* = 17) compared to control patients without infections (Non-infection, NI, *n* = 20). The two groups were matched by age, body mass index (BMI), and the invasiveness of the surgical procedure. Peripheral blood mononuclear cells (PBMCs) were collected after surgery and subjected to high-dimensional mass cytometry.

**Results:**

Cluster analysis identified 46 immune cell clusters, 30 of which exhibited significant differences between the I and NI patients. Natural killer cells as well as myeloid dendritic cells were decreased in I patients compared to NI patients (*p*=0.0269; and *p*=0.0267, respectively). Conversely, Th17, CD69^+^ and HLA-DR^+^ CD4^+^ T cells were significantly increased in I patients compared to NI patients (*p*=0.0422, *p*=0.0267; *p*=0.0267, respectively). ROC curve analysis of immune cell counts demonstrated potential for differentiating NI from I patients.

**Discussion:**

This study reveals that patients with SSI exhibit a significantly altered immune cell profile, with greatest differences observed in increased HLA-DR^+^ CD4^+^ T cells and decreased numbers of several innate immune cells. The diagnostic potential of these markers may prove clinically relevant, and further research into the impact of activation and exhaustion may yield future therapeutic strategies.

## 1 Introduction

Spinal surgical site infections (SSIs) remain a critical concern in clinical practice due to the significant impact they have on patient outcomes and healthcare expenditures. Across various spine procedures, infection rates vary substantially, ranging from approximately 1% to over 10% depending on the complexity of instrumentation, underlying comorbidities, and surgical technique (1, 2). A specific and challenging subset of these infections is implant-associated vertebral osteomyelitis, which can develop when bacterial biofilms form on spinal instrumentation, severely complicating both the initial diagnosis and subsequent treatment (3, 4). Most SSIs occur within 12 weeks after index surgery, with *Staphylococcus epidermidis* and *Staphylococcus aureus* being the most commonly detected pathogens (5). The management of these infections typically requires biofilm-active antibiotic therapy, potentially with complex revision surgeries. This imposes an enormous physical and psychological burden on patients and substantial overall healthcare costs (6, 7).

Despite aggressive interventions, persistent or recurrent infection can occur, emphasizing the importance of robust preventative strategies (8, 9). Factors such as immunocompromised states, advanced age, high body mass index, diabetes mellitus, or other comorbidities are associated with heightened susceptibility to infection (10, 11). Moreover, the type of spine surgery (major or minor) is a determining factor. Major surgery, such as lumbar fusion may involve extensive disruption to muscles and bones, requiring more complex management of tissue and the use of implants to ensure structural integrity. In contrast, minor surgery, such as sequestrectomy typically employs smaller incisions, minimizing trauma to tissues and reducing recovery impact. Importantly, major surgeries carry a higher risk of postoperative infections, with rates increasing (2.4-8.5%) due to the greater surgical trauma, longer operative times, and frequent use of instrumentation, whereas minor surgeries generally have lower infection risks due to their less invasive nature (1-2%) (12).

Localized infection of the spine differs in several important respects from other musculoskeletal infections such as fracture-related infections (FRI) or periprosthetic joint infection (PJI) (13). Primarily this is due to the anatomy of the spine, its close relationship with the nervous system and the varying presence of haematopoietic bone marrow. Typically, spinal infections affect vertebral bones and intervertebral discs, posing a risk for neurological complications such as spinal cord compression and related symptoms (14). Beyond neurological compromise, infections of the spine can precipitate structural instability through endplate destruction, implant loosening, pseudarthrosis and vertebral collapse, leading to segmental kyphosis and spinal deformity that may require complex revision surgery (15, 16).

Definitive diagnosis of SSI may require pathogen identification from intra-operative biopsy culture, as non-invasive techniques may lack sensitivity, particularly in low grade infections (17, 18). Diagnosis typically relies on a combination of clinical suspicion, laboratory inflammatory markers such as C-reactive protein (CRP), microbiological cultures from intraoperative or wound specimens, and imaging studies (19). The detection of SSIs at an early stage allows for timely antimicrobial intervention with the possibility of implant retention, potentially reducing the need for extensive revision surgery and improving prognosis. Given the high morbidity associated with these infections, continuous efforts to refine prevention, identification, and treatment strategies remain a priority in spinal surgery care (20–23).

The value of blood biomarkers in diagnosing spinal infections remains relatively limited, partly due to the variability in the markers studied and the lack of standardized cutoff values (24). Common inflammatory markers such as CRP, white blood cell (WBC) count, and erythrocyte sedimentation rate (ESR) are frequently used to suggest infection, but their sensitivity and specificity can vary widely depending on the context (24, 25). Some studies indicate that CRP and WBC are useful in monitoring response to treatment, yet their effectiveness in early diagnosis or differentiation from non-infectious postoperative changes is limited (25). In scenarios with low-virulent pathogens, and when the distinction between infection, aseptic pseudarthrosis, or delayed wound healing is required, the diagnostic value of CRP and leukocyte count is markedly limited (26).

A deeper understanding of the host immune response in SSIs may yield clinically relevant insights into pathogenesis, enable the identification of novel diagnostic biomarkers, and inform future approaches to prevention, risk prediction, and targeted therapy. High-dimensional analytic techniques, such as mass cytometry, provide unprecedented resolution into immune cell phenotypes, including activation and exhaustion markers, which could improve the accuracy of diagnostic biomarkers (27). In this prospective study, we systematically examine the immune cell profiles of patients who developed SSIs following spine surgery compared to matched controls. Our aim is to describe the immunological changes during SSI in comparison to patients without SSIs.

## 2 Methods

### 2.1 Patient cohort

Patients undergoing lumbar spine surgery at the Schulthess Clinic (Zürich, Switzerland) were consecutively screened for enrolment into two groups and prospectively included: the SSI group (Group I: Patients who developed a postoperative spinal surgical site infection) and the control group (Group NI: Patients with uneventful recovery). All participants had to be at least 18 years old, capable of understanding the study information, and willing to provide written informed consent. For the definition and diagnosis of SSI, the Centers for Disease Control and Prevention (CDC) and National Healthcare Safety Network (NHSN) criteria were applied. Control patients were matched to the SSI group by procedure type, age, sex, and body mass index. Exclusion criteria included malignancy, systemic autoimmune or inflammatory disorders, existing infectious diseases, haematological (myelo)proliferative conditions (e.g., leukaemia), major trauma, and cervical spine interventions. Under these criteria, we aimed to include 40 patients—20 in each group. Blood samples were taken as soon as the infection was established, in average 18.8 days after the surgery. A schematic illustration of the study can be found in the supplements (**Supplementary Figure 10**).

### 2.2 Surgical and clinical management

Only patients undergoing lumbar spine surgery were included. Surgical procedures were classified *a priori* based on the performed intervention rather than by a composite invasiveness score. Minor surgery was defined as lumbar decompression without instrumentation and was performed using a minimally invasive surgical (MIS) approach. Major surgery was defined as lumbar fusion with instrumentation and was performed via a conventional posterior midline approach; all fusion procedures were transforaminal lumbar interbody fusions (TLIF), and the number of fused levels was documented for each patient. Cervical and thoracic procedures were excluded. Blood samples in the infection group were obtained at the time of clinical diagnosis of SSI, prior to the initiation of empiric antibiotic therapy. Superficial and deep infections were not explicitly differentiated; in the study group´s institutional clinical practice, the diagnosis of an SSI mandates surgical revision, and accordingly all infected patients underwent a DAIR procedure (debridement, antibiotics, irrigation, and retention of implants), resulting in a clinically homogeneous infection cohort. This was an observational study, and standard clinical management was not altered at any time, except for the collection of additional blood samples for study purposes.

### 2.3 Ethical considerations and data handling

All patients provided written informed consent prior to participation. Blood sample and data collection and processing were approved by the Swiss Ethics Committee (BASEC #2020-00783) and conducted in accordance with the Declaration of Helsinki. Raw data were securely stored at the Schulthess Clinic Zurich, and only pseudonymized datasets were used for data analysis.

From October 27^th^, 2020, to May 30^th^, 2024, we prospectively enrolled 42 spine surgery patients, from which 5 patients failed inclusion criteria, leaving 17 I patients and 20 NI patients for analysis. Patients included in the study were matched based on sex, age, body mass index (BMI), and smoking or diabetes status (**Table 1A**). The I cohort’s mean age was 63.4 ± 15.7 years, with 47% females. The mean BMI was 28.0 ± 4.2 kg/m^2^. Median last pre-operative CRP was 4.4 ± 3.6 mg l^-1^, rising to 125.4 ± 113.5 mg l^-1^ (IQR) in the first post-operative sample. Surgical complexity ranged from single-level decompressions (n = 12) to multilevel fusions (n = 4, including n =2 two and three level each), with a median of 1 operated level. The mean blood loss was 378.7 ± 252.7 ml, and the mean surgery time was 223.9 ± 95.0 min. In n = 5 cases, the surgery was categorized as “minor” and in n = 12 cases as “major”. SSI emerged after a median of 19.5 ± 10.5 days (5–48 days); isolates were predominantly *Staphylococcus aureus* (n = 10) and coagulase-negative staphylococci (n = 2), *Escherichia coli* (n = 2), streptococci (n = 1) and n = 2 otherwise categorized pathogens (**Table 1B**); no episode progressed to culture-positive sepsis. Prior to the revision surgery the inflammatory activity was low in most infected patients, with a mean CRP of 6.0 ± 5.9 mg/l. The first post-operative sample demonstrated a pronounced inflammatory surge, with CRP rising to 99.9 ± 100.1 mg/l. Leukocyte counts were within the normal range before surgery (mean 8.07 ± 2.7 G/l) and increased moderately post-operatively (11.9 ± 4.5 G/l) (**Table 1C**).

**Table 1:** Patient recruitment, group assignment and clinical data.

A		
Group (number of patients)	NI (20)	I (17)
% female	40%	47.06%
Minor surgical procedure	6	5
Major surgical procedure	14	12
Age	63.3 ± 15.2	63.4 ± 15.7
BMI	27.3 ± 4.3	28 ± 4.2
Diabetes patients	10%	29%

B		
Pathogen	Patients (out of 17)	
S. aureus	10 (59%)	
S. epidermidis	2 (12%)	
Streptococci	1 (6%)	
E. coli	2 (12%)	
Others	2 (12%)	

C		
Group (number of patients)	NI (20)	I (17)
Pre surgery CRP	2.5 ± 3.3 mg/l	6.0 ± 5.9 mg/l
Post surgery CRP	-	99.9 ± 100.1 mg/l
Pre surgery leukocyte count	7.2 ± 1.9 G/l	8.07 ± 2.7 G/l
Post surgery leukocyte count	-	11.9 ± 4.5 G/l

(A) Summary of the clinical data of patients included in the study. (B) Pathogens found in spine infection patients. (C) CRP and leucocyte count before and after (only I) surgery in all patients.

Over the same period, we enrolled 20 non-infected controls. Controls were individually matched to infected patients for age, sex, and surgical magnitude. Their mean age was 63.3 ± 15.2 years with 40% females, and mean BMI of 27.3 ± 4.3 kg/m^2^. Peripheral blood samples were taken at the same day, prior to elective surgery and at average 50.4 ± 14.3 days postoperatively. The median pre-operative CRP measured 2.5 ± 3.3 mg l^-1^. Leucocyte counts were in the normal rage measured preoperatively (mean 7.2 ± 1.9 G/l). The surgical spectrum mirrored that of the infected cohort—n = 14 single-level surgeries, n = 6 multilevel/long-segment constructs (n = 5 2-level, n = 1 3-level) with a median of 1 operated level. The mean estimated blood loss was 326.0 ± 161.7 ml and the mean surgery time was 189.0 ± 86.3 minutes. In n = 6 case the surgery was categorized as “minor” (decompression) and in n = 14 cases as “major” (fusion) (**Table 1C**).

### 2.4 Isolation of peripheral blood mononuclear cells

Peripheral blood samples were collected in the morning under standardized conditions using 10 ml EDTA Vacutainer Tubes (BD, USA). In the study group, postoperative blood was drawn following clinical diagnosis of spinal implant-associated infection, prior to revision surgery. In the control group, samples were collected postoperatively. All samples were shipped to the AO Research Institute Davos using an overnight courier and arrived within 24 hours of collection. Samples were processed on the day of arrival at the Institute.

PBMCs were isolated from blood by density gradient centrifugation. The blood was diluted with an equal volume of phosphate buffered saline (PBS, SIGMA #P4417)) and slowly layered on to 15 ml density gradient medium (Histopaque 1077, SIGMA #10771). After density gradient centrifugation for 25 min at 800 x g at room temperature (RT), the resulting PBMC layer was transferred to a new centrifugation tube and washed twice with PBS (10 min centrifugation at 300 x g at RT). The PBMC pellet was resuspended in alpha-MEM and the number of PBMCs was determined using a cell counting device.

For cryopreservation, the counted cells were resuspended in cold freezing medium (10% DMSO/45% FCS/45% alpha-MEM) at a cell concentration of 5–20 x 10^6^ cells per millilitre. The aliquots were then frozen in a Mr. Frosty freezing container (Thermo Fisher Scientific, USA) overnight at -80 °C and cryopreserved in the gas phase of liquid nitrogen until further use.

### 2.5 Antibody labelling and titration

Metal conjugation of antibodies with lanthanide and cadmium isotopes was carried out using the Maxpar Antibody Labeling Kit (Fluidigm, San Francisco, CA, USA) following the manufacturer’s instructions. After conjugation, cadmium antibodies were eluted in W-buffer (Fluidigm) containing HRP-Protector™ peroxidase stabilizer, and lanthanide antibodies were eluted in W-buffer (Fluidigm) containing antibody stabilizer buffer (Candor Bioscience) supplemented with 0.05% sodium azide. Antibody labelling with metal tags was performed using the MaxPAR antibody conjugation kit (Fluidigm) according to the manufacturer’s instructions. The final volume of each antibody preparation was measured, and an equal volume of antibody stabilizer buffer (Fluidigm; supplemented with 0.05% sodium azide or HRP-Protector) was added prior storage at 4 °C. All conjugated antibodies were subsequently titrated using PBMCs from healthy donors to determine the optimal working concentrations.

### 2.6 Antibody staining

Cryopreserved PBMCs were thawed and stained for mass cytometry in several batches with the immune profiling kit (Standard biotools, San Francisco, CA, USA) and additional conjugated antibodies. The metal-conjugated antibodies used for single-cell mass cytometry are listed in **Supplementary Table 1**.

PBMCs were incubated with Human TruStain FcX (BioLegend) for 10 min at RT to block Fc receptors (FcR). After centrifugation (300 x g, 5 min), the cells were stained with metal-conjugated antibodies for 30 min at RT (**Supplementary Table 3**). After staining and washing by Maxpar^®^ Cell Staining Buffer (CSB) (Fluidigm) two times (300 x g, 5 min), cells were incubated with 1.6% FA solution (Thermo Fisher Scientific™) for 10 min at RT. After centrifugation (800 x g, 5 min), cells were incubated in 250 μM Cell-ID™ intercalator-Ir (Fluidigm) in Maxpar Fix and Perm Buffer (Fluidigm) overnight at 4 °C. The next day, cells were centrifuged (800 x g, 5 min), resuspended in 100 µl remaining volume and frozen at -80 °C until the day of acquisition.

### 2.7 Data acquisition

Cells in Maxpar Intercalator solution were washed twice with CSB followed by two washes with cell acquisition solution (CAS) (Fluidigm) right before acquisition. Samples were filtered using a Polystyrene tube with 35 µm cell strainer Cup, and EQ Four Element Calibration Beads (Fluidigm) were added to the suspension. EQ bead normalization was performed using Fluidigm’s standard passport-based algorithm on raw.fcs files from each acquisition day. The procedure identifies EQ beads via Ce140Di/Eu151Di/Eu153Di/Ho165Di/Lu175Di signature, with channel-specific factors computed by passport/observed median ratios (linearly interpolated) and applied to non-bead events prior to bead removal. Cells were acquired on a Helios™ (Fluidigm). The event rate was between 250–300 events per second. Data were normalized after acquisition using EQ Four Element Calibration Beads (Fluidigm) in each experiment. Acquisition was performed on 8 different days using patient samples from different groups each day to avoid batch effects (**Supplementary Figure S9**).

### 2.8 Data processing and analysis

Data was analysed using FlowJo software V10 (Treestar, Ashland, OR, US) and graphically displayed using GraphPad Prism 10 (GraphPad Software Inc., La Jolla, CA, US). A gating strategy that includes all distinguishable cell types can be found in the supplements (**Supplementary Figures S4**-**S8**) and was adapted from Maxpar Pathsetter software for CyTOF data. Statistics were generated using GraphPad Prism 10. Statistics for comparison of NI *vs* I were performed using Mann-Whitney test or Welch`s test, depending on normality distribution which was tested beforehand. P-values <0.05 were considered significant and are indicated in the graphs. P-values were adjusted for multiple comparisons using the Benjamini–Hochberg correction.

Normalized data was uploaded to OMIQ (Boston, MA, USA) and an arcsinh transformation with a coefficient of 5 was applied. A standardized cleanup strategy was performed which was followed as described by Bagwell et al. (28): Further downstream analysis and reporting of data used the transformed data. Data were checked for consistency over time and non-conforming sections were gated out using flowCut (Boston, MA, USA). Viable CD45+ single cells were selected by manual gating. For lineage populations analysis, total live CD45+ single cells were down sampled to 10,000 events per subject for analysis followed by unsupervised uniform manifold approximation and projection (UMAP). UMAP was performed using the following parameters (neighbours= 15; minimum distance= 0.4; components= 2; metric= Euclidean; embedding initialization= spectral) followed by PhenoGraph clustering (k = 100). Data was further analysed with EdgeR (OMIQ, Boston, MA, USA) to determine significantly different clusters, or by generating heatmaps with Euclidean clustering. Welch’s t-test on log2-transformed metacluster proportions was performed to assess differences in metacluster proportions (the volcano plot), and Benjamini-Hochberg- (BH-)adjustment was applied to the resulting P-values. Immunological parameters distinguishing NI from I were evaluated using receiver operating characteristic (ROC) curve analysis in GraphPad Prism (GraphPad Software Inc., La Jolla, CA, US). Optimal cut-off values, sensitivity, and specificity were determined using the maximum Youden index. A ROC curve combining two predictors was generated based on binary logistic regression predictive probabilities.

## 3 Results

PBMCs of control patients (NI) and patients with surgical site infections (I) were subjected to high dimensional immune phenotyping mass cytometry (43-color), exploring innate and adaptive immune cell populations and activation and exhaustion status. Analysis of baseline characteristics such as age and percentage of females revealed no significant statistical differences across the groups (**Table 1**).

### 3.1 T cell clusters differ between infected and non-infected patients

**Figure 1:**
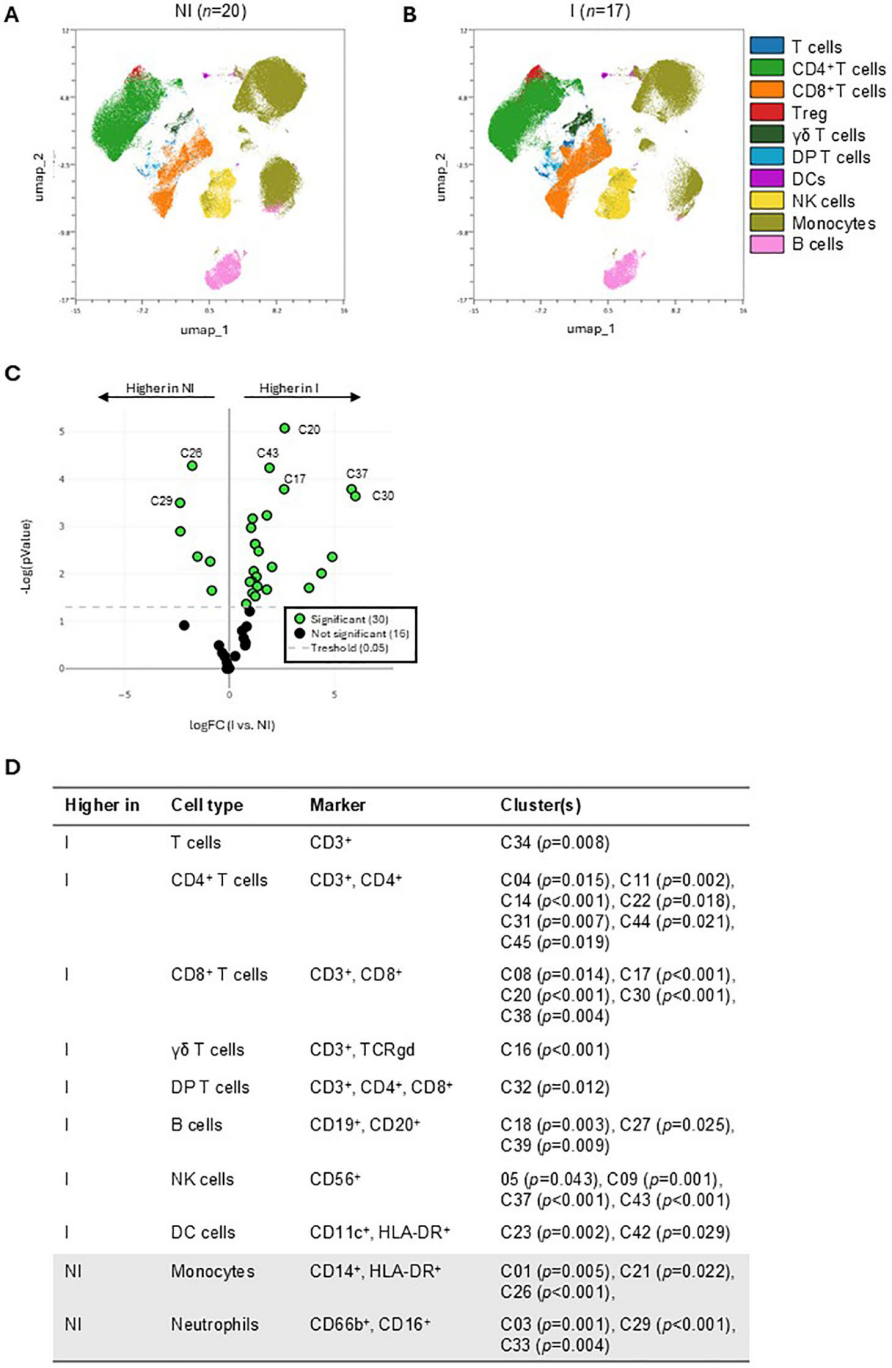
Uniform manifold approximation and projection (UMAP) of all PBMCs in NI and I patients. UMAP representative of all cells from **(A)** NI and **(B)** I patients coloured by lineage. **(C)** Volcano plot for the comparison of all 46 metaclusters (MC) (as proportions of total number of cells per subject) between I and NI patients. The X-axis depicts the difference in means of the log2-transformed proportion of each MC; the Y axis depicts the -log10-transformed Benjamini-Hochberg- (BH)-adjusted P-value obtained using Welch’s t-test. Green coloured points above the horizontal line represent significantly upregulated or downregulated metaclusters in NI *vs* I patients. **(D)** Table of significantly different clusters and their corresponding cell types and markers.

UMAP representation of all PBMCs from NI (**Figure 1A**) and I patients (**Figure 1B**) illustrates the most abundant cell types in the two study groups. The UMAPs indicate that NI patients displayed an apparent greater abundance of monocytes compared to I patients (**Figures 1A, B**). These NI patients concurrently displayed a lower abundance of T cells, specifically T regulatory cells, Double positive (DP) T cells, γδ T cells, CD8^+^ T cell and CD4^+^ T cell clusters than NI patients (**Figures 1A, B**). The UMAP representative NI and I patients coloured by clusters are shown in the supplement (**Supplementary Figure 11**).

Unsupervised clustering analyses were subsequently performed using PhenoGraph and edgeR to interrogate these abundances in detail, revealing 46 metaclusters, of which 30 significantly differed between I and NI patients (**Figure 1C**). In agreement with the UMAP representation, a number of T cell subsets were significantly less abundant in NI patients compared to I patients, including DP T cells (C32), γδ T cell (C16), CD4 T cells (C04, C11, C14, C22, C31, C44, C45) and CD8^+^ T cells (C08, C17, C20, C30) cluster (**Figure 1D**). A comparatively lower number of clusters were identified that were more abundant in NI patients, specifically monocytes (C01, C021, C26) and neutrophils (C03, C29, C33) (**Figure 1D**).

### 3.2 Innate and adaptive immune cells show significant differences between I and NI patients

**Figure 2:**
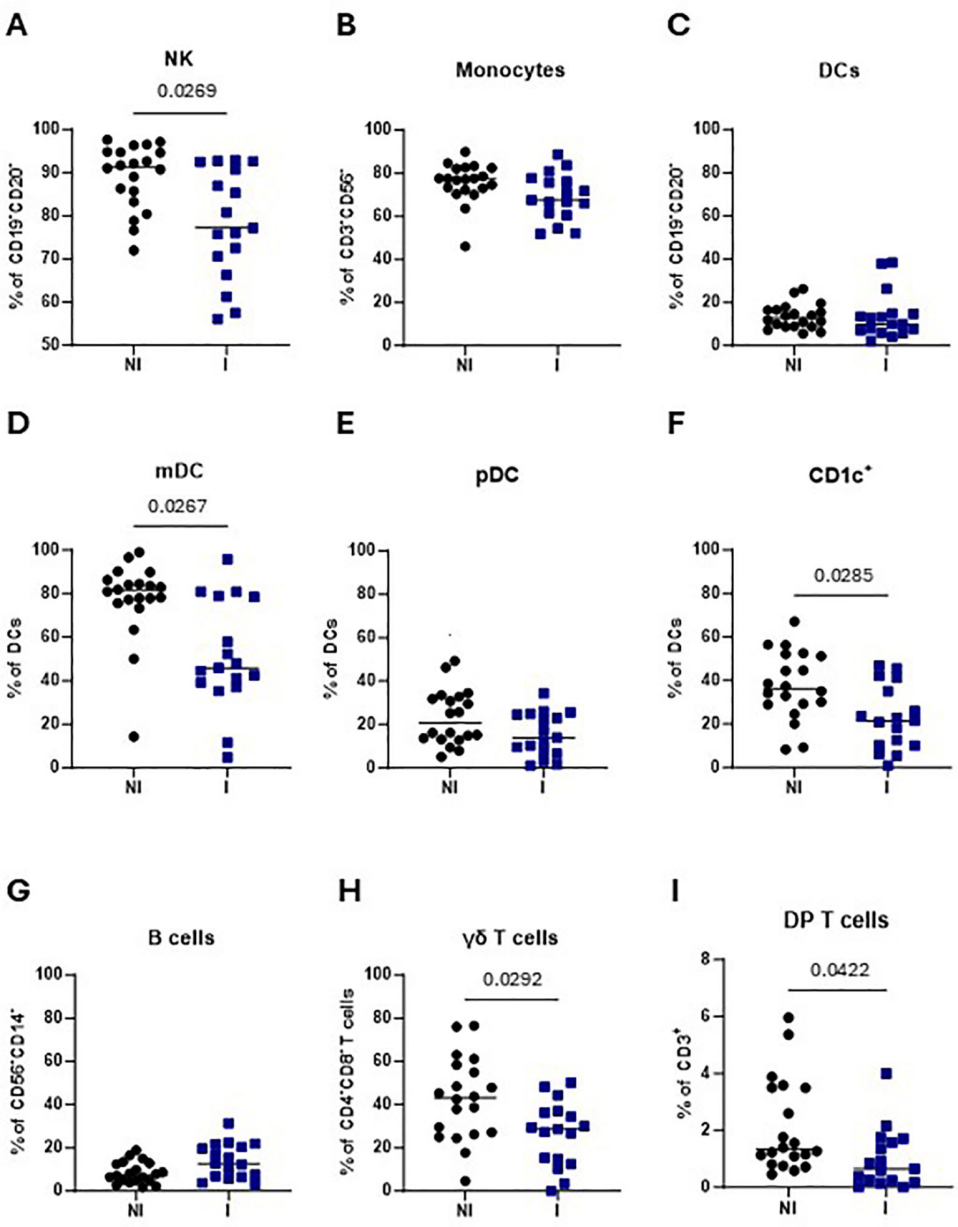
Innate and adaptive immune cells in I and NI patients. **(A)** NK cells, **(B)** monocytes, **(C)** DCs, **(D)** mDCs, **(E)** pDCs, **(F)** CD1 expression on DCS **(G)** B cells, **(H)** γδ T cells and **(I)** DP T cells in NI compared to I patients. Data shown are from individual patients (NI: *n* = 20; I: *n* = 17). Statistical analyses performed using Mann-Whitney test or Welch`s test.

Since the cluster analyses revealed significant differences in abundance in immune cell populations, they were investigated at the individual patient level between NI and I patients (**Figure 2**).

In accordance with the UMAP analysis, I patients displayed decreased natural killer (NK) cells (*p* = 0.0269) compared to NI patients (**Figure 2A**). Although the total dendritic cells (DCs) count were not different between the groups (**Figure 2C**), mDCs (*p* = 0.0267) (**Figure 2D**), as well as CD1^+^ DCs (*p* = 0.0285) (**Figure 2F**) were all decreased in I compared to NI patients. Moreover, I patients showed decreased γδ T cells (*p* = 0.0292) and DP T cells (*p* = 0.0422) compared to NI patients (**Figures 2H, I**). Monocytes, pDCs and B cells were not significantly different between the groups (**Figures 2B, E, G**). The differences seen between cluster and gating results arise because cluster analysis incorporates marker intensities to define subpopulations within each cell type, whereas conventional gating groups all marker-positive cells together regardless of intensity.

### 3.3 Infection leads to alterations in CD8^+^ T cell activation profile

**Figure 3:**
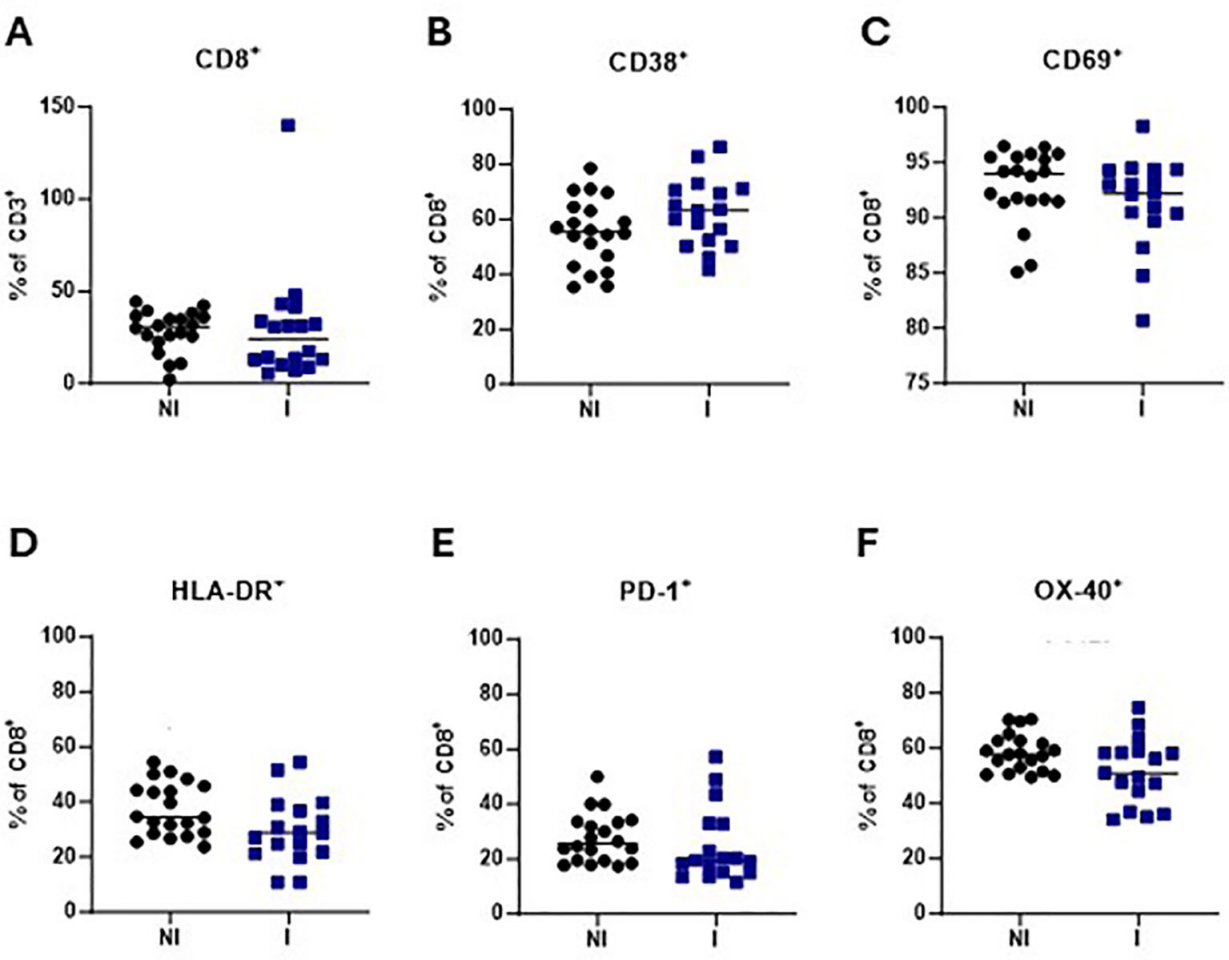
CD8^+^ T cells and activation markers in NI compared to I patients. **(A)** CD8^+^ T cells, **(B)** CD38^+^, **(C)** CD69^+^, **(D)** HLA-DR^+^, **(E)** PD-1^+^ and OX-40^+^ CD8^+^ T cells in NI compared to I patients. Data shown are from individual patients (NI: *n* = 20; I: *n* = 17). Statistical analyses performed using Mann-Whitney test or Welch`s test.

Given the significant differences in the T-cell populations observed in the cluster analysis, we proceeded to a more detailed investigation of CD8^+^ T cells in NI and I patients. There were no significant differences in CD8^+^ T cells between the groups (**Figure 3A**). For further characterization, the activation status of CD8^+^ T cell was examined, investigating the activation markers CD69, CD38 and HLA-DR on CD8^+^ T cells. NI and I patients showed no significant differences in CD38^+^ (**Figure 3B**), CD69^+^ (**Figure 3C**), HLA-DR^+^ CD8^+^ T cells (**Figure 3D**), PD-1^+^ (**Figure 3E**) and OX-40^+^ (**Figure 3F**) CD8^+^ T cells.

### 3.4 Infection induces changes in the activation profile of CD4^+^ T cells

**Figure 4:**
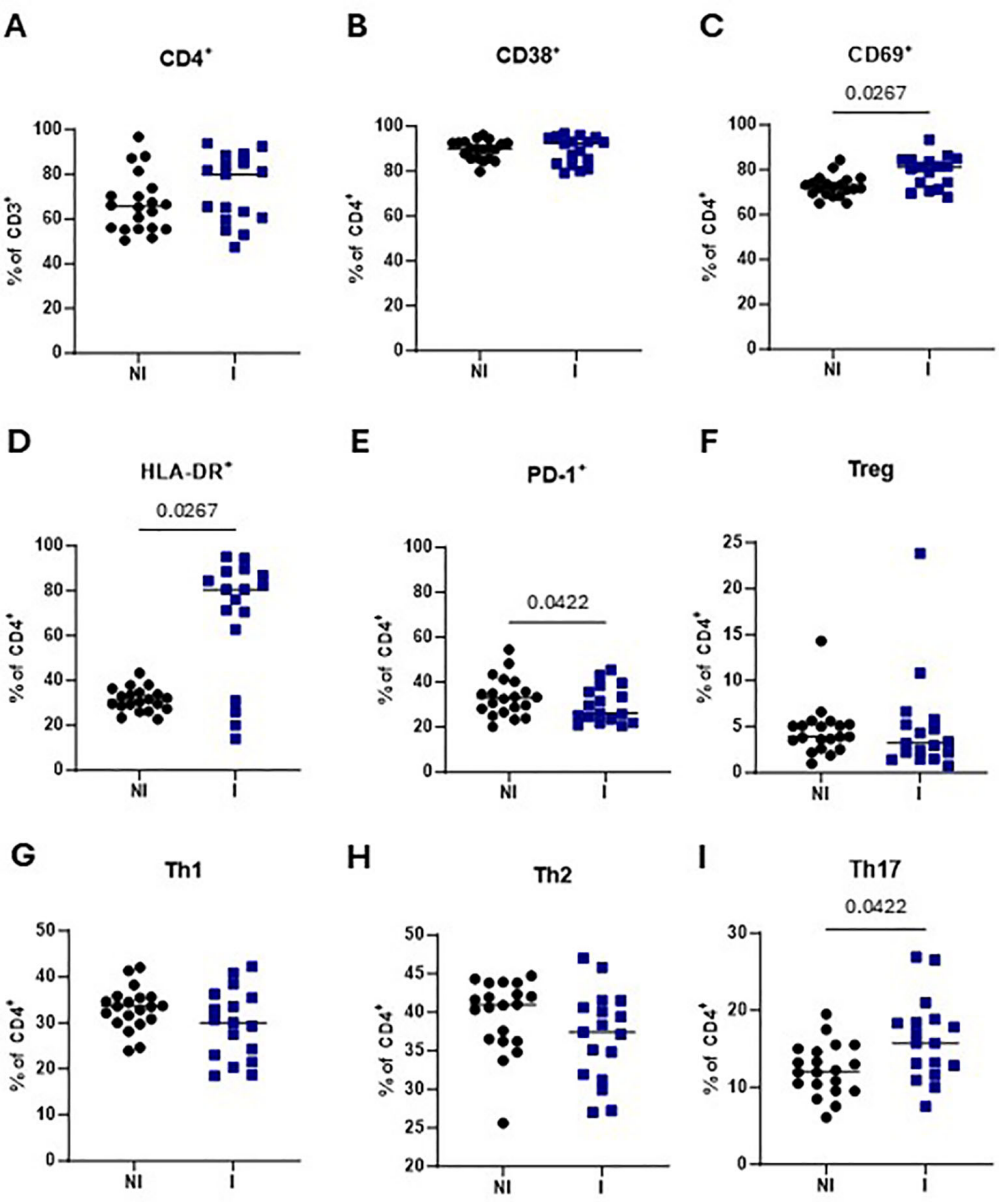
CD4^+^ T cells and activation markers in NI compared to I patients. **(A)** CD4^+^ T cells, **(B)** CD38^+^, **(C)** CD69^+^, **(D)** HLA-DR^+^ and **(E)** PD-1^+^ CD4+ T cells in NI compared to I patients. **(F)** Treg, **(G)** Th1, **(H)** Th2 and **(I)** Th17 cells in NI compared to I patients. Data shown are from individual patients (NI: *n* = 20; I: *n* = 17). Statistical analyses performed using Mann-Whitney test or Welch`s test.

Similar to the analysis of CD8^+^ T cells (**Figure 3**), further analysis was performed to get a deeper insight in the phenotype of CD4^+^ T cells in NI and I patients. NI and I patients showed no significant differences in CD4^+^ T cells (**Figure 4A**) and CD38^+^ CD4^+^T cells (**Figure 4B**).

However, I patients had significantly increased CD69^+^ (*p* = 0.0267) (**Figure 4C**), HLA-DR^+^ (*p* = 0.0267) (**Figure 4D**) and decreased PD-1^+^ (*p* = 0.0422) (**Figure 4E**) CD4^+^ T cells compared to NI patients. NI and I patients showed no significant differences in Treg (**Figure 4F**), Th1 (**Figure 4G**) and Th2 cells (**Figure 4H**). I patients had significantly increased Th17 cells (*p* = 0.0422) compared to NI patients (**Figure 4I**).

### 3.5 ROC curves of significantly different immune cells show diagnostic potential

**Figure 5:**
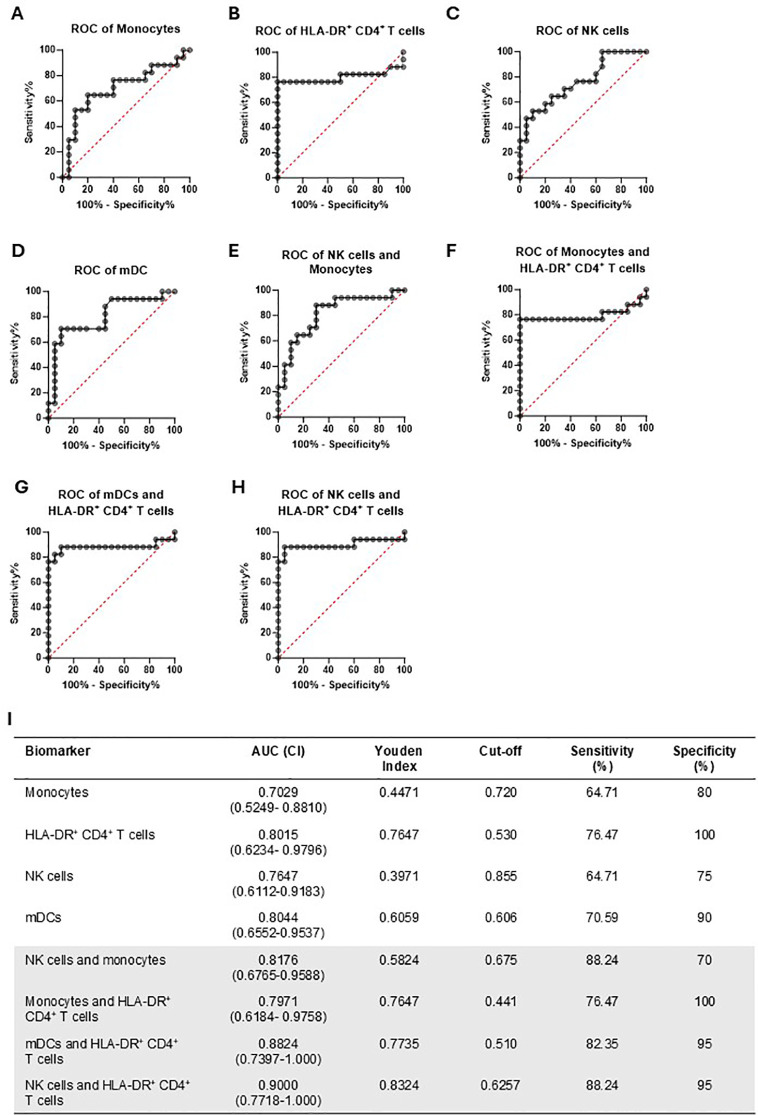
Receiver operating characteristic curves. **(A)** ROC curve for the discrimination of NI and I based on the number of **(A)** monocytes, **(B)** HLA-DR^+^ CD4^+^ T cells, **(C)** NK cells and **(D)** mDCs, their combination **(E-H)**. **(I)** Table corresponding to the graphs to each marker as well including the area under the ROC curve, the optimal cut-off at the maximum Youden index with the corresponding sensitivity and specificity for each biomarker (unshaded) and their combination (shaded in grey).

Given the distinct variations in NK cells, monocytes, HLA-DR^+^ CD4^+^ T cells as well as mDCs we next assessed their potential as biomarkers of infection. Receiver Operating Characteristic (ROC) analysis assessed the diagnostic potential of most significantly different immune cells (**Figures 5A–D**) as well as their combinations (**Figures 5E–H**) to distinguish NI from I patients. The areas under the curve (AUC) and the Youden index are shown in **Figure 5I** for each of the ROC curves and for their combination.

The best performing cell marker was HLA-DR^+^ CD4^+^ T cells with an area under the curve (AUC) of 0.8015, Youden index was 0.7647 with a sensitivity of 76.47% and a specificity of 100% (**Figure 5I**). Combined analysis revealed the best performing combination were mDCs and HLA-DR^+^ CD4^+^ T cells showing an AUC of 0.8824 and a Youden index of 0.7735 with a sensitivity of 82.35% and a specificity of 95% as well as the combination of NK cells and HLA-DR^+^ CD4^+^ T cells showed an AUC of 0.9000 and a Youden index of 0.8324 with a sensitivity of 88.24%, and a specificity of 95%.

## 4 Discussion

SSIs are among the most concerning postoperative complications associated with spinal procedures. Even with improvements in surgical techniques, sterilization methods, and antibiotic prophylaxis, SSIs remain a significant risk that can delay recovery, cause severe complications, and increase the need for further medical intervention. This study demonstrates that patients who develop localized SSIs display distinct alterations in immune cell profiles in peripheral blood. These findings and the identified markers may hold relevance for diagnosing infection or immune-targeting interventional strategies.

When comparing patients with SSI to those without, one of the most notable differences observed was a relatively broad reduction in innate immune cell types in the peripheral blood of infected patients. Patients who developed SSIs had significantly lower levels of NK cells and dendritic cell populations in peripheral blood compared to matched controls at the time of infection detection. Innate immune cells mediate early cytotoxic defence (NK), migrate to inflammation sites, differentiate to clear pathogens and repair tissue (monocytes/macrophages), and initiate adaptive immunity through antigen presentation (DCs). Moreover, NK cells produce interferon-γ (IFN-γ), which is critical for the control of viral and intracellular pathogen infections. Peripheral blood levels of innate immune cells have been shown to be reduced in certain infections, particularly during acute systemic responses such as sepsis. Monocytes and NK cells are rapidly recruited from circulation to sites of infection (29–31). However, these reductions are not consistent or permanent in patients with localized infections. Transient elevations in innate cell counts can occur during the acute phase, especially in severe infections like sepsis (30, 32, 33). As infections progress to chronic or deep-seated stages, increased recruitment of these cells into tissues may lead to relative decreases in circulating levels. The magnitude and direction of these changes are influenced by infection severity, anatomical location, and the balance between peripheral recruitment and haematopoietic replenishment (29, 30, 32–40). Notably, detailed evaluations of these dynamics specifically in SSIs are scarce in the literature, highlighting a gap in our understanding in that context. It remains to be determined if these SSIs differ from other infections in how they affect peripheral blood innate immune cell counts, or whether these patients already have innate immune cell deficiencies that predispose them to infection. The histopathological appearance of spinal surgery related infection is poorly described in the literature, but neutrophil and monocyte accumulation in the affected tissues would be expected, similar to what has been reported in periprosthetic joint infection and osteomyelitis (41). These innate immune cell alterations may also be influenced by the surgical procedure. Müller-Heck et al. demonstrated that invasive spine surgery can induce a selective suppression of NK-cell mediated IFN-γ responses in peripheral blood while monocyte induced interleukin (IL)-12 activity remains preserved, indicating an early innate vulnerability specific to surgical trauma rather than infection. The preservation of monocyte IL-12 activity is characteristic of surgical stress, not the typical immune activation seen in infection, where both monocyte and IL-12 responses are usually upregulated (40). Moreover, in addition to other innate immune cells, monocyte counts in peripheral blood often decline early after surgery due to stress-induced redistribution, followed by a compensatory increase as part of the normal immune response to tissue injury or return to baseline after spinal cord injury (31, 39, 42). Notably, while monocyte numbers may decrease transiently, their IL-12 activity can remain preserved, reflecting intact functional capacity despite numerical changes. The innate immune alterations found in this study may reflect rather a secondary response to ongoing SSI than a response to surgery. The consistent reduction in NK cells, monocytes, and DC subsets in the infected group, but not in the not infected group that had similar operative procedures, suggests a systemic immune profile that is altered due to infection.

The other major finding of the study was the reduced numbers of numerous T cell subsets in infected patients and significant changes in certain activation/exhaustion markers. A reduction in γδ T cells and double-positive (CD4^+^CD8^+^) T cells in the infected group point to broader immune alteration, particularly in unconventional T cell subsets that are usually considered important for early mucosal defence and immune modulation. Lower γδ T cell numbers have previously been shown to impair infection clearance, as mouse studies show delayed bacterial clearance and reduced survival in the absence of these cells (43). Specifically, γδ T-cells are innate-like lymphocytes that provide a rapid source of IL-17 and IFN-γ at wounded tissue interfaces (44, 45) which is needed for Th1 and Th17 cell responses. Th17 cells rapidly expand and are essential for controlling bacterial infections, as well as helping to bridge innate and adaptive immunity, especially during early immune responses. Increased Th17 cells have also been shown to be elevated in patients with bacterial infections, and is a classical response to infection (46, 47).

Although CD4^+^ and CD8^+^ T cell counts were not different between groups, activation and exhaustion markers revealed significant changes. We observed an increased number of CD69^+^ and HLA-DR^+^ CD4^+^ T cells, which indicates activation and proliferation of these cells. HLA-DR^+^ CD4^+^ T cells are primed effector memory cells and typically associated with high cytokine output (IFN-γ, IL-2). This suggests that spine SSI leads to a detectable change in activation of CD4^+^ T cells that are activated to support pathogen control locally in the spine (48). However, it was not mirrored in CD8^+^ T cells. This selective activation may indicate a stronger T helper cell response, which may also promote the expansion of Th17 cells, given the cytokine environment typically associated with activated CD4^+^ T cells during inflammation (39). Notably, Th17 cell expansion is consistently observed in prosthetic joint infection, osteomyelitis, and sepsis, reflecting their critical role in driving inflammation and tissue pathology during these infections (49–51).

Persistent HLA-DR expression together with higher expression of PD-1 could reflect ongoing antigen stimulation and could signal the onset of T cell exhaustion, a hallmark of chronic immune activation in persistent infections or inflammation (52). However, the decreased number of PD-1^+^ CD4^+^ T cells in infected patients suggests a state of active T cell activation and recent antigen exposure. While PD-1 is typically associated with T cell exhaustion, its lower expression in this context may reflect a shift toward effector or memory differentiation, or a reduction in chronic antigen stimulation, potentially indicating a more responsive rather than exhausted CD4^+^ T cell pool during acute infection.

Our stratified analysis of immune cell abundance revealed distinct immunological profiles between I and NI patients, depending on surgical severity (**Supplementary Figures 1**, **S2**). The distinction between major and minor spine surgery is crucial due to differences in complexity, tissue involvement, and invasiveness and these may mask or accentuate the impact of infection on immune responses. The magnitude of surgery itself did not meaningfully alter the systemic immune profile once patients had reached the later postoperative phase. Within the infected and non-infected group, few significant differences in immune clusters were observed between minor and major surgery types, indicating that systemic immune alterations at the time of sampling were driven predominantly by infection rather than by surgical extent. Clinically, this suggests that while major surgery is intuitively expected to impose greater immunologic stress, these early postoperative effects may largely normalize during the recovery period, such that infection becomes the dominant determinant of systemic immune status.

ROC curve analysis of immune cell counts demonstrated potential for differentiating NI from I patients. While the individual markers hold some potential for distinguishing between groups, they lack strong sensitivity and specificity. They are unlikely to be clinically useful on their own; however, the combination of two biomarkers performed substantially better, yielding a noticeably higher AUC and a more favourable balance of sensitivity and specificity. As such, the paired marker model may hold greater clinical or research utility than either marker alone, especially in addition to CRP, WBC and ESR as these markers are non-specific, variably sensitive, and often insufficient on their own to reliably distinguish infection from non-infectious inflammation. This limitation is evident in studies showing that conventional markers have only moderate sensitivity (45–81%) and specificity (40–80%) for infection, highlighting the need for more precise immune profiling tools (53). Due to the small sample size, the ROC curve findings should be considered preliminary. Therefore, independent validation of the ROC curve in a larger cohort will be required before any clinical application can be considered. Additional analysis assessed whether elevated CRP levels in patients are associated with changes in immune cell composition or activation, highlighting the potential influence of systemic inflammation on immune responses. However, no correlation between high or low cell numbers based on the CRP value was found (**Supplementary Figure 3**).

This study highlights immune changes in patients with SSI, but limitations should be noted. To begin with, the analysis was based on a relatively small sample size (n=37), so confirming findings with a larger cohort would be of value. Nevertheless, the data generated in this study includes a deeper profile and larger cohort than the existing literature. Moreover, the study focused on characterizing immune cell types without assessing their functional activity. The combined alterations in NK cells, dendritic cell subsets, and CD4^+^/CD8^+^ T-cell activation would prompt the use of functional assays such as cytokine release or intracellular cytokine staining, proliferation assays and degranulation/cytokine assays to determine whether these phenotypic changes reflect functional impairment. Future research that incorporates functional assessments, would provide a more complete picture of the involvement of the immune system in the pathogenesis of SSI. In addition, to note is that our monocyte data in UMAP clusters did not fully match the FlowJo gating strategy. While useful for visualizing broader patterns, UMAP is less precise for defining monocyte subsets due to its reliance on dimensionality reduction. Subtle differences in marker expression can be observed in the UMAP, leading to an overlap between closely related cell populations. Therefore, the FlowJo gating strategy remains more reliable for accurate gating and subset definition. Another point is that we used a general TCR γδ cell antibody to define γδ T cells and did not use a more specific TCR Vδ1 and Vδ2 antibody. For future studies and more detailed examination of γδ T cells, additional antibodies should be included. One more point is that postoperative sampling time between groups differed. Blood samples in infected patients were obtained at the time of clinical diagnosis (median 19 days), reflecting the natural occurrence of early SSIs in this cohort, whereas controls were sampled later during routine follow-up after uneventful recovery. Postoperative immune responses are dynamic and may confound comparisons. However, perioperative immune-monitoring studies show that sterile surgery-induced immune alterations are predominantly early, with substantial recovery within days to one week in uncomplicated courses. Longitudinal single-cell analyses demonstrate normalization of major immune populations by postoperative day 3, with some dendritic-cell and NK-cell subsets remaining altered up to day 7 (54). Persistent immune alterations have been associated with complications rather than uncomplicated recovery (55, 56). Nevertheless, time-dependent effects cannot be fully excluded, and longitudinal, time-matched studies are needed to clearly distinguish surgical from infection-driven immune changes. Finally,we acknowledge that dedicated metal-minus-one (MMO) controls were not performed for the CyTOF panel and positivity thresholds were determined empirically using unstimulated biological control patients (NI).

This work highlights the host immune response in implant-associated spinal infections, a complex clinical challenge with significant morbidity. Notably, SSI was linked to a distinctive immune signature, characterized by marked depletion of innate cells, including NK cells, monocytes, mDCs and pDCs, alongside heightened CD4^+^ T cell activity and blunted CD8^+^ T cell co-stimulation. Moreover, the paired marker ROC model may offer greater clinical value, particularly when combined with conventional markers such as CRP, WBC, and ESR.

## Data Availability

The original contributions presented in the study are included in the article/**Supplementary Material**. Further inquiries can be directed to the corresponding author.
