# Supplementary material for: Systemic immune responses in patients with spine surgery related infections
Source: Front Immunol. 2026 May 26;17:1805889. doi: 10.3389/fimmu.2026.1805889 (PMC13246327; doi:10.3389/fimmu.2026.1805889)
Supplement: Supplementary file 1 [file DataSheet1.docx]

# Supplements

The following table lists the antibodies used. For self-conjugated antibodies as well as antibodies bought from Standard biotools the concentration was titrated and is indicated in the table.

Table S1: Antibodies used.

The following table lists the raw p values and the corrected p values after Benjaminin Hochberg multiple hypothesis correction.

Table S2: p values.

**Infection is the primary driver for changes between NI and I patients in minor and major surgery type**

A comparison of immune profiles was performed to determine the influence of surgery type (minor vs. major) on differences between NI and I patients.

UMAP representation of all PBMCs from minor (Figure S1A) and major (Figure S1C) surgery type in NI, and minor (Figure S1B) and major (Figure S1D) surgery in I patients illustrates the significant clusters in the two study groups.

Unsupervised clustering analyses were subsequently performed using PhenoGraph and edgeR to interrogate these abundances in detail, revealing 46 metaclusters, of which 8 and 27 significantly differed in minor surgery type between I and NI patients (Figure 6E). Moreover, 46 metaclusters were identified, of which 8 significantly differed in major surgery type between I and NI patients (Figure S1F).

Only some clusters changed in comparison between NI and I patients when including the aspect of surgery type. In minor surgery, a greater abundance of B cell cluster C35 and a lower abundance of B cell cluster C41 was found in I than in NI patients (Fig. S1A, B and E). For the major surgery type, we found a higher abundance in CD4^+^ T cell cluster C13, NK cell clusters C38 and C43 as well as DC clusters C23 and C42 in I than in NI patients (Fig. S1C, D and F).


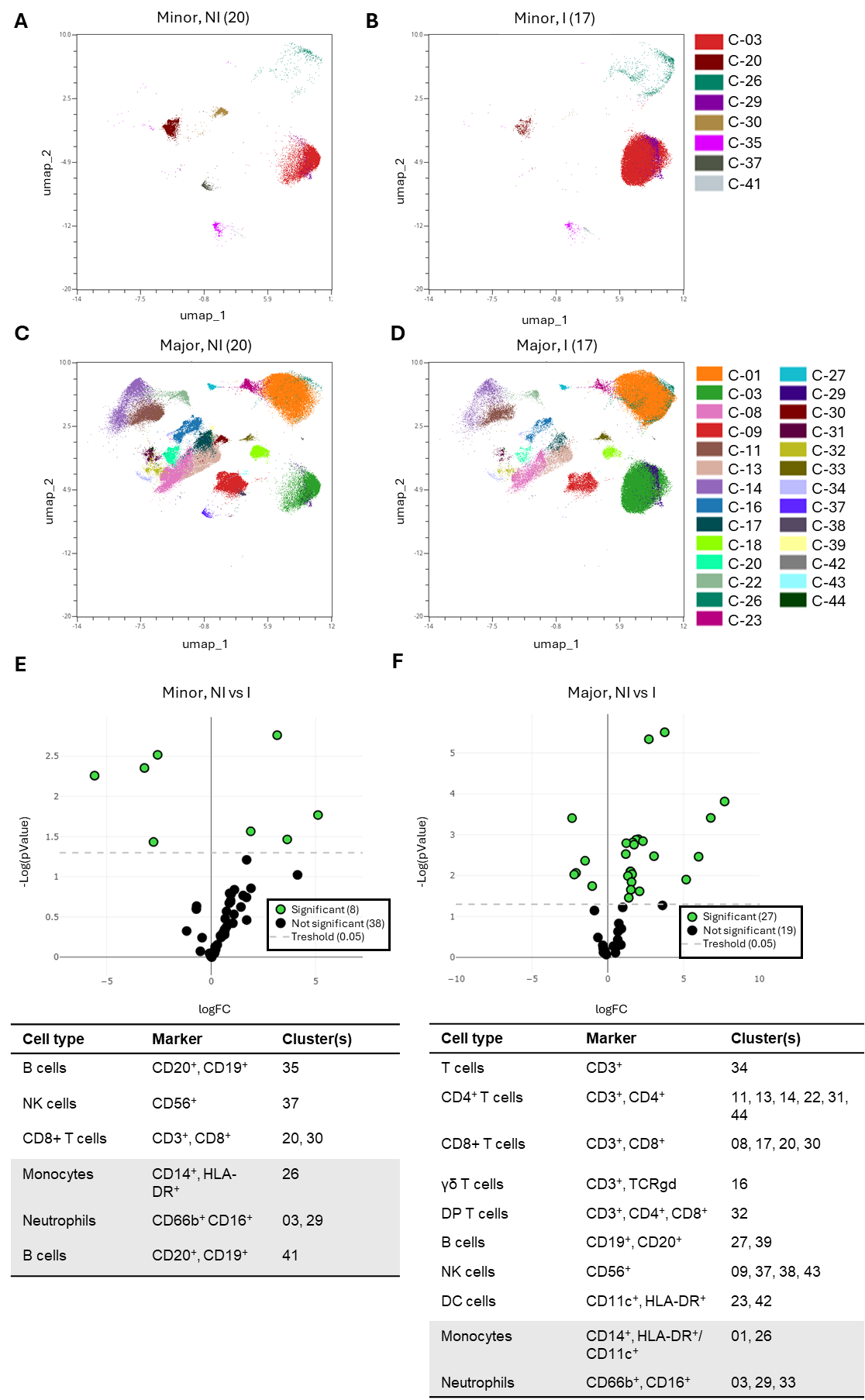


Figure S1: Uniform manifold approximation and projection (UMAP) of all I and NI patients PBMCs comparing minor and major surgery type. UMAP representative of all significantly different clusters from minor surgery type in A) NI and B) I patients and from major surgery type in C) NI and D) I patients. Volcano plot for the comparison of all 46 metaclusters (MC) (as proportions of total number of cells per subject) between I and NI patients for E) minor and F) major surgery type with table of significantly different clusters and their corresponding cell types and markers. The X-axis depicts the difference in means of the log2-transformed proportion of each MC, the Y axis depicts the -log10-transformed Benjamini-Hochberg- (BH)-adjusted P-value obtained using Welch’s t-test. Green coloured points above the horizontal line represent significantly differentially abundant metaclusters.

**Comparison of immune cell clusters between minor and major surgery types within NI and I patients revealed minimal changes within patient groups**

To investigate if the surgery type shows differences in immune cells within NI and I patients, major and minor surgery type were compared. Unsupervised clustering analyses were subsequently performed using PhenoGraph and edgeR to interrogate these abundances in detail, revealing 46 metaclusters, of which only 1 significantly differed in minor versus major surgery type in NI patients (Figure S2A). In I patients, 46 metaclusters were identified, of which 6 significantly differed in minor versus major surgery (Figure S2B).


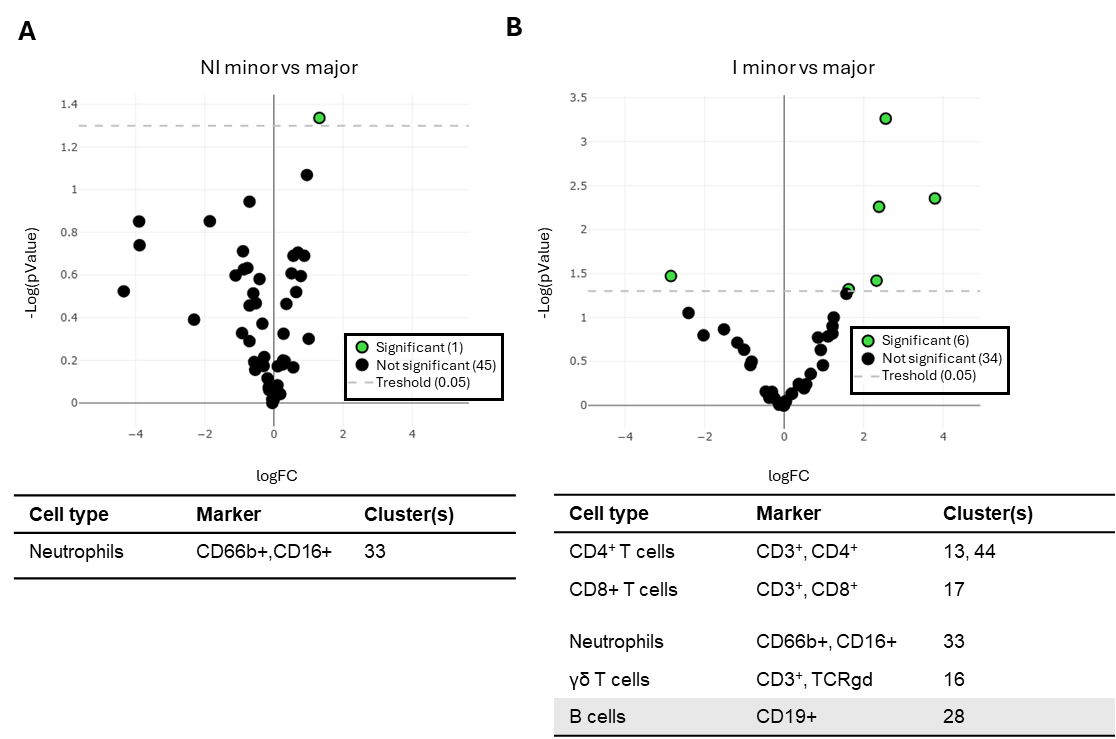


Figure S2: Comparison of minor vs major surgery type in NI and I patients. Volcano plot for the comparison of all 46 metaclusters (MC) (as proportions of total number of cells per subject) between minor and major surgery type in A) NI and B) I patients with table of significantly different clusters and their corresponding cell types and markers. The X-axis depicts the difference in means of the log2-transformed proportion of each MC, the Y axis depicts the -log10-transformed Benjamini-Hochberg- (BH)-adjusted P-value obtained using Welch’s t-test. Green coloured points above the horizontal line represent significantly differentially abundant metaclusters.

**CRP values of infected patients and their distribution in immune cells**

C-reactive protein (CRP) is a liver-derived acute-phase protein that reflects systemic inflammation. Blood CRP levels were measured to assess inflammatory responses. To evaluate whether elevated CRP levels correlate with our findings, patients were separated into those with higher CRP values (highlighted in red, Figure S3). Patients with elevated CRP levels were distributed across the range of values for all markers, indicating no clear separation or unique immune profile associated with high CRP status.


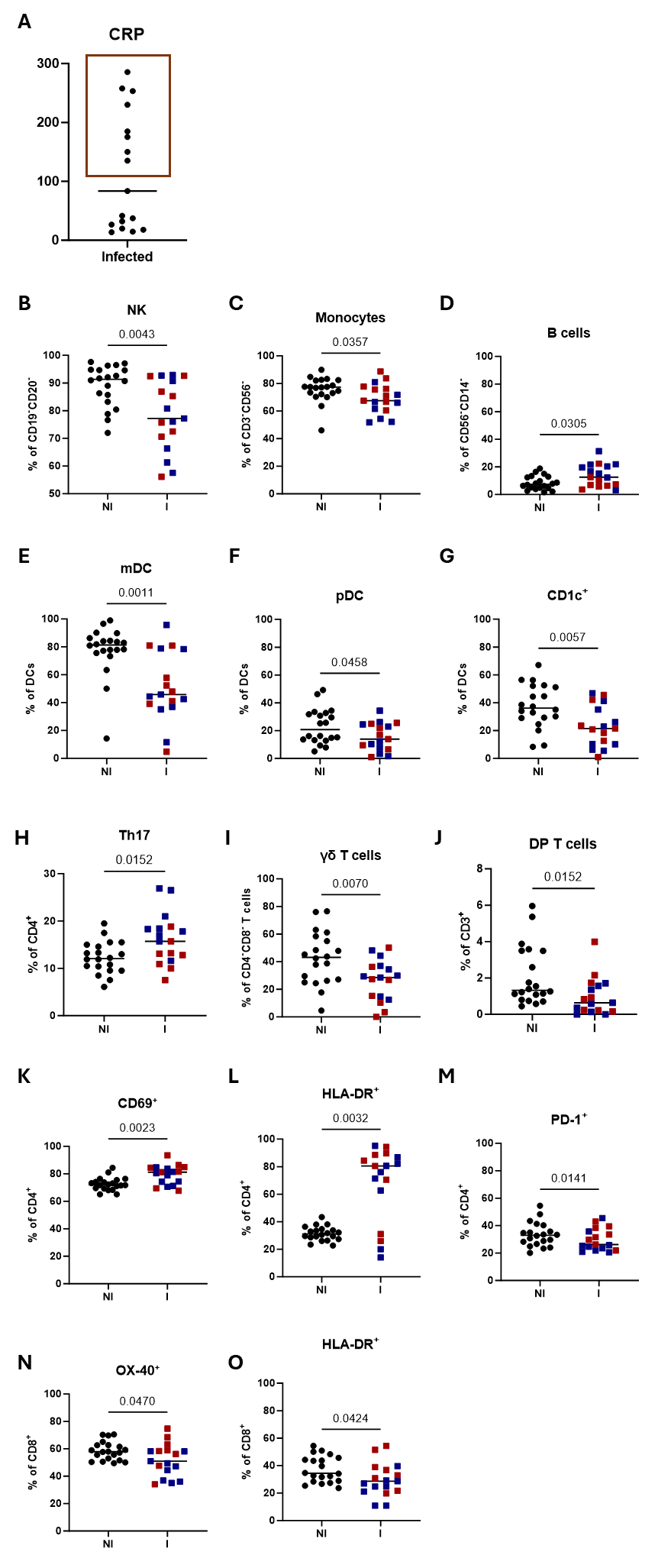


Figure S3: CRP values and innate and adaptive immune cells in I and NI patients. A) CRP values of patients, B) NK cells, C) monocytes, C) B cells, E) mDCs, F) pDCs, G) CD1^+^ DCS, H) Th17cells, I) γδ T cells, J) DP T cells K) CD69^+^, L) HLA-DR^+^, M) PD-1^+^ CD4^+^ T cells as well as N) OX-40+ and O) HLA-DR^+^ CD8^+^ T cells in NI compared to I patients. Data shown are from individual patients (NI: *n*=20; I: n=17). Statistical analyses performed using Mann-Whitney test or Welch`s test. CRP high patients are marked in red.

**Gating strategy**

Figure S4 depicts the gating strategy of mass cytometric data for all PBMCs. All representative scatter plots presented are obtained from peripheral blood of NI patients. After cleaning of the files with MaxparPathsetter software to get the beads excluded, the files were analysed using FlowJo software V10. First, the remaining beads were excluded, and it was checked if all the cells were positive for DNA (DNA-1 against DNA-2). The next step was to exclude dead cells (DNA-1 against Live-Dead). CD45^+^CD66b^-^ and CD66^+^ cells were gated on viable cells. On CD45^+^CD66b^-^ cells which are no B cells were gated (CD19 against CD20). T cells, monocytes, DCs and NK cells can be gated on no B cell gates. Moreover, cells that are not B cells were excluded out of CD45^+^CD66b^-^ cells (CD56 against CD14). B cells were gated on B cells-1 (CD19 against CD3). Naïve and memory B cells were gated (CD19 against CD27). Plasmablasts were gated on memory B cells (CD38 against CD20). Eosinophils (CD16^-^CD294^+^) and Neutrophils (CD16^+^CD294^-^) were gated on CD66b^+^ cells.

**
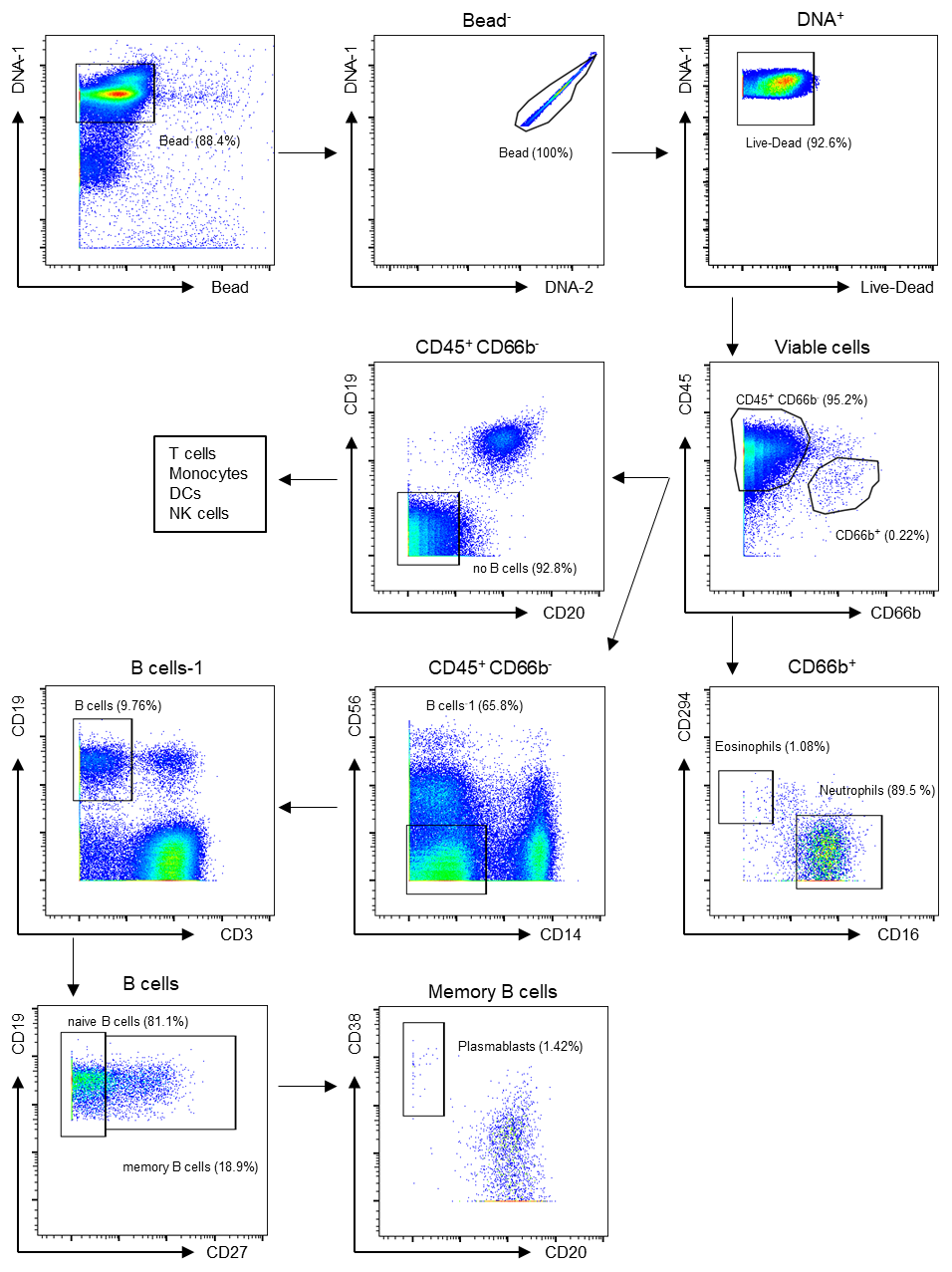
**

Figure S4: Gating strategy 1: Lymphocyte gating

The gate no B cells was used to distinguish NK and DCs (CD3^-^CD14^-^), CD3^-^CD56^-^ cells (CD3^-^CD56^‑^) and no monocytes (CD56^-^CD3^-^) (Figure S5A). Figure S5B shows the gating strategy of NK cells. First, CD45RA^+^CD123^-^ cells were gated on NK and DCs. Afterwards NK cells (CD56^+^) were distinguished and divided in early (CD56^+^CD57^-^) and late (CD56^+^CD57^+^) NK cells. Figure S5C shows the gating strategy of DCs. DC (CD56^-/+^HLA-DR^+^) were gated on NK and DCs. pDCS (CD123^+^CD11c^-^) and DCs (CD123^-^CD11c^+^) were gated on DC. mDCs (CD11c^+^CD38^+^) were gated on DCs.


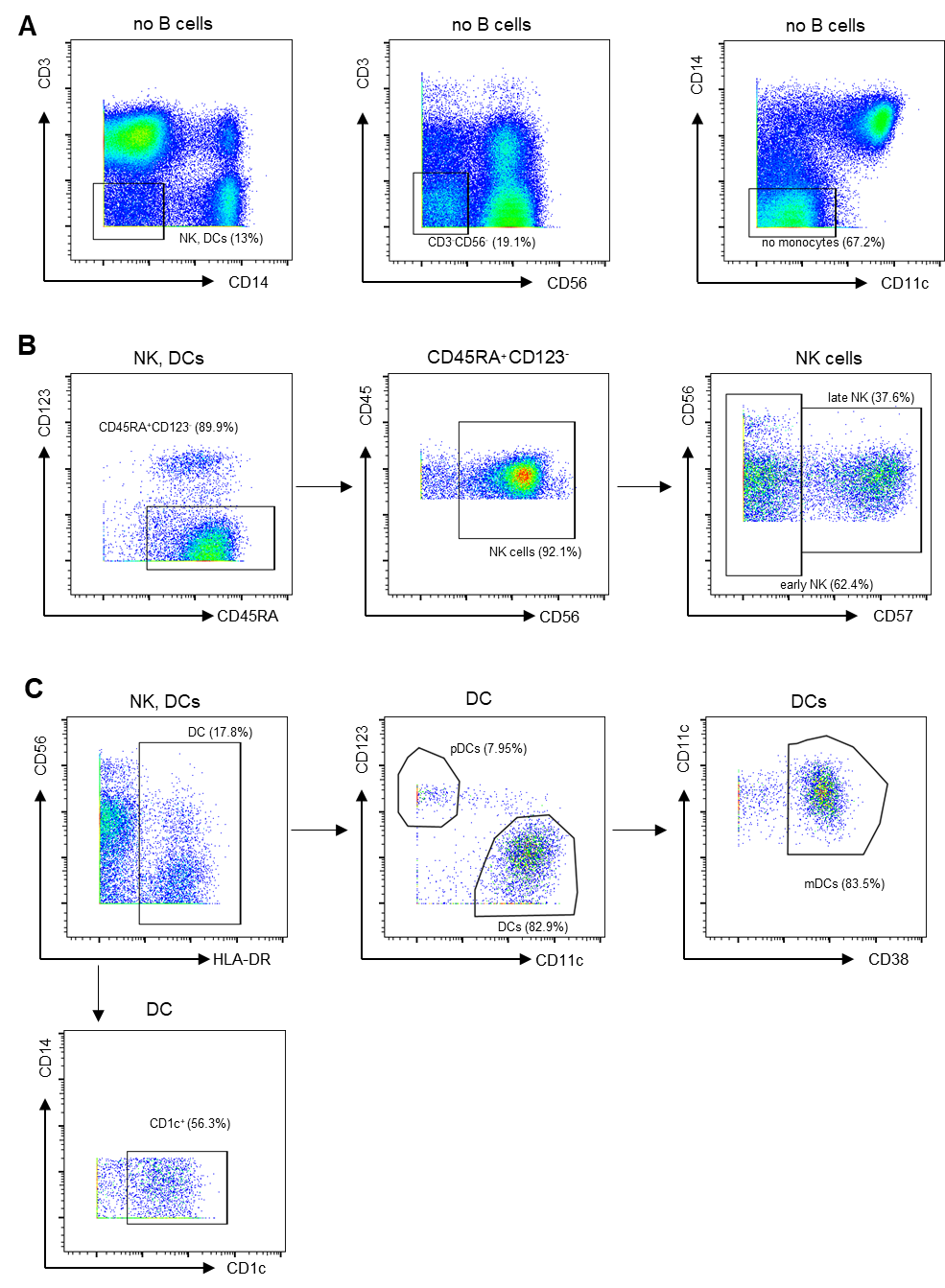


Figure S5: Gating strategy 2: NK and DCs


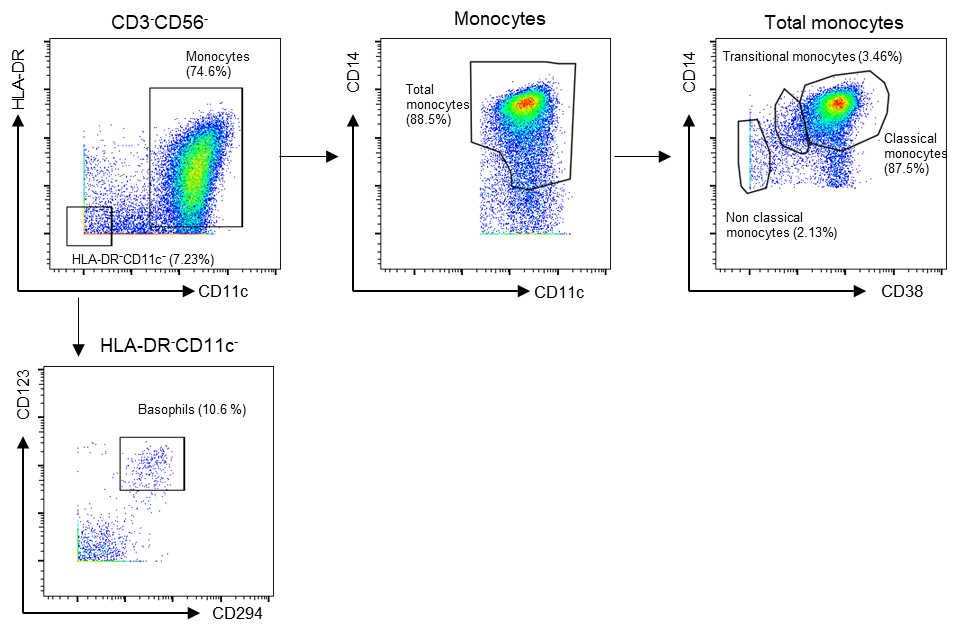
Figure S6 shows the gating strategy of monocytes and basophils. Monocytes (CD11c^+^HLA^-^DR^low/+^) were gated on CD3^-^CD56^-^ cells. CD14^-^ cells were excluded and classical (CD38^+^CD14^+^), non-classical (CD38^low^CD14^+^) and transitional monocytes (CD38^-^CD14^low^) were gated. HLA^-^DR^-^CD11c^-^ cells (HLA-DR^-^CD11c^-^) were gated on CD3^-^CD56^-^ cells. Basophils (CD123^+^CD294^+^) are gated on HLA^-^DR^-^CD11c^-^ cells.

Figure S6: Gating strategy 3: Monocytes

T cells (CD3^+^CD45^+^) were gated on no monocytes. γδ T cells were excluded (TCRγδ^-^) and on αβ T cells the markers CD4 and CD8 were used to distinguish between CD4^+^ and CD8^+^ T cells (Figure S17A). CD25^-^ cells were gated on CD4^+^ T cells and T helper cells were gated; Th17 (CCR6^+^CXCR3^-^), Th2 (CCR6^-^CXCR3^-^), Th1 (CCR6^-^CXCR3^+^) and Tfh (CCR6^+^CXCR3^+^) (Figure S17B).The differentiation status was checked using the markers CCR7 and CD45RA; naïve cells (CD45RA^+^CCR7^+^), effector memory cells (CD45RA^-^CCR7^-^), central memory cells (CD45RA^-^CCR7^+^) and terminal effector cells (CD45RA^+^CCR7^-^) (Figure S7C). Figure S7D shows the different activation marker gating: CD38^+^, CD69^+^ and HLA^-^DR^+^ CD4^+^ T cells as well as the exhausted cells gating PD-1^+^ CD4^+^ T cells. Figure S7E shows the gating strategy for Treg cells. CD4^+^ T cells which are CCR4^+^ were used to distinguish CD45RA^-^CD45RO^+^ cells. Treg (CD25^+^CD127^-^) were gated on CD45RA^-^CD45RO^+^ cells.


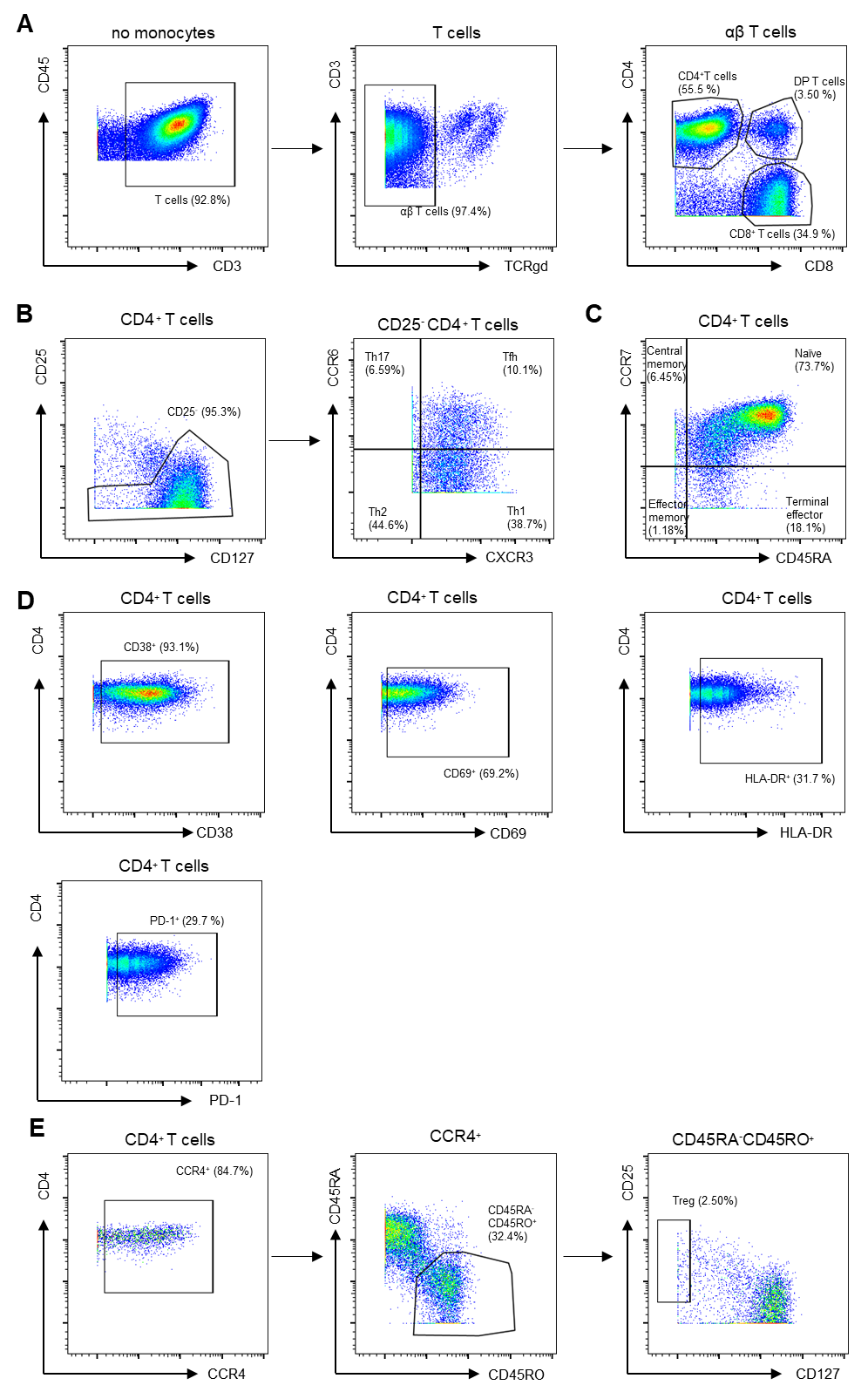


Figure S7: Gating strategy 4: CD4^+^ T cells


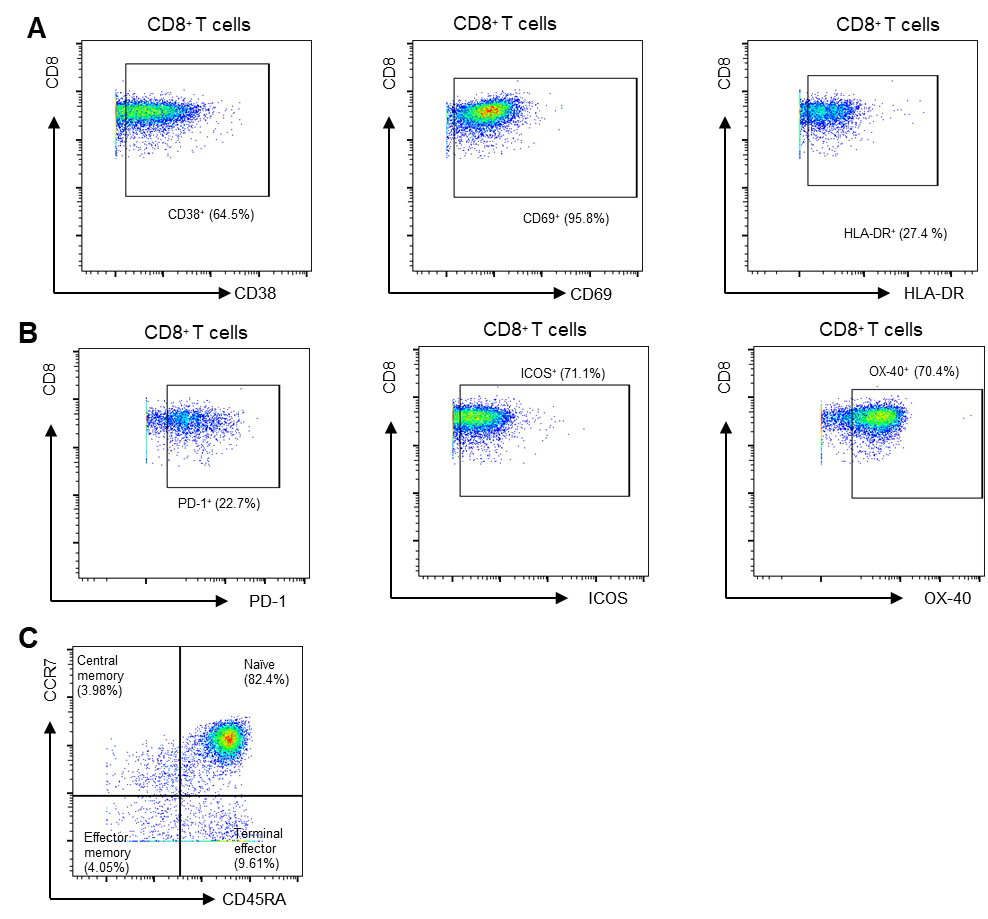
Figure S8A shows the different activation marker gating: CD38^+^, CD69^+^, and HLA-DR^+^ on CD8^+^ T cells. Figure S8B shows the exhaustion marker gating PD-1^+^, ICOS^+^, and OX-40^+^ on CD8^+^ T cells. The differentiation status was checked using the markers CCR7 and CD45RA; naïve cells (CD45RA^+^CCR7^+^), effector memory cells (CD45RA^-^CCR7^-^), central memory cells (CD45RA^-^CCR7^+^), and terminal effector cells (CD45RA^+^CCR7^-^) (Figure S8C).

Figure S8: Gating strategy 5: CD8^+^ T cells


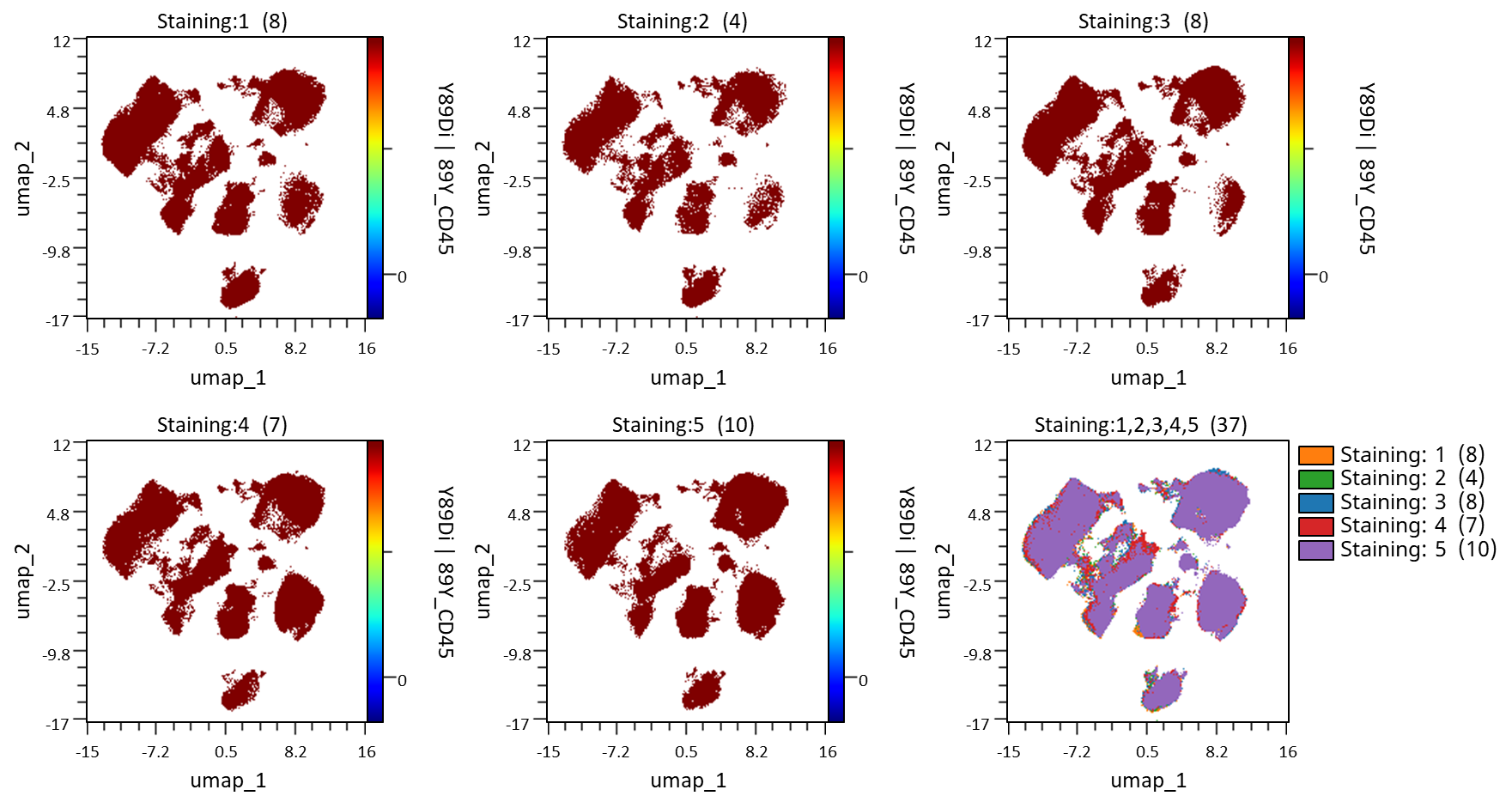


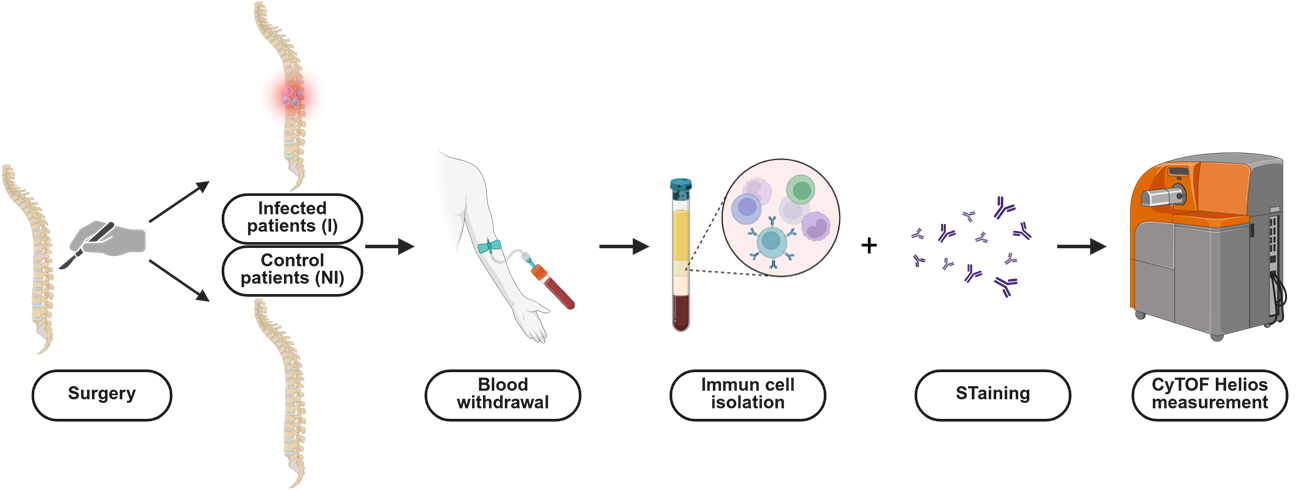
Figure S9: Batch effect of staining days

Figure S10: Schematic illustration of study


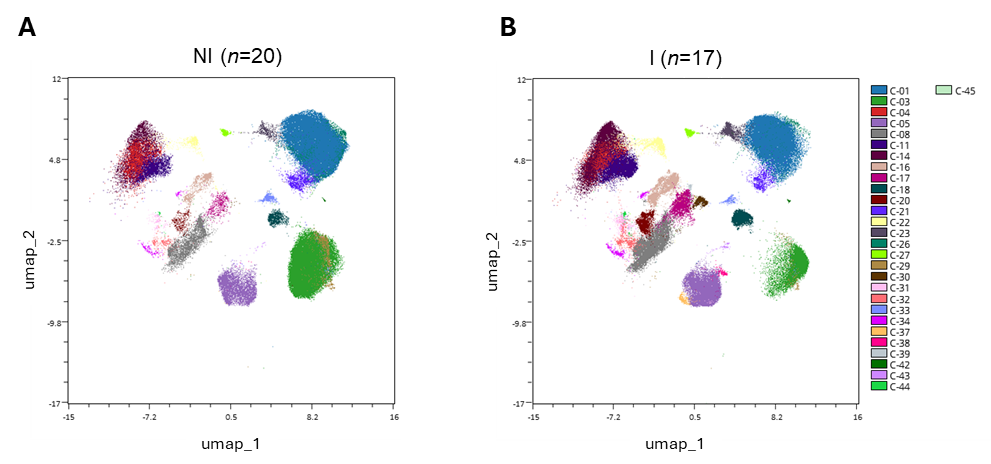


**Figure S11: UMAP of all PBMCs in NI and I patients. UMAP representative of all cells from A) NI and B) I patients coloured by cluster.**
